# Sigmoid Colon Cancer Masked by Refractory Diverticulitis With Abscess

**DOI:** 10.7759/cureus.48326

**Published:** 2023-11-05

**Authors:** Akiyoshi Ikebata, Shuji Mikami, Jae H Yoo, Shinichi Tsuwano, Shigeo Hayatsu

**Affiliations:** 1 Department of Surgery, National Hospital Organization Saitama Hospital, Saitama, JPN; 2 Department of Pathology, National Hospital Organization Saitama Hospital, Saitama, JPN

**Keywords:** case report, colon cancer, colorectal cancer, refractory, diverticulitis

## Abstract

Colorectal cancer (CRC) can occasionally coexist with diverticulitis, thereby complicating diagnosis and treatment. In cases of refractory diverticulitis, it is important to consider the possibility of malignancy and determine appropriate treatment strategies. An 85-year-old male presented with lower left abdominal pain; he was admitted for further examination and the treatment of suspected sigmoid diverticulitis. On examination, a firm mass was palpated in the lower left quadrant. Imaging revealed sigmoid diverticulitis, partial abscess formation, and the involvement of the small bowel and abdominal wall. Although malignancy was suspected, a definitive diagnosis was not made. Because of the refractory nature of the disease, early surgical intervention, sigmoid colectomy, partial small bowel resection, abdominal wall resection, and lymph node dissection, was performed in accordance with the malignancy protocol. Pathologic diagnosis revealed adenocarcinoma within the diverticulitis with negative resection margins, indicating curative surgery. The low preoperative diagnostic rate of CRC associated with diverticulitis highlights the need for vigilance. Refractory diverticulitis may indicate the presence of concealed malignancy requiring surgical intervention. In the management of refractory diverticulitis, it is important to consider the potential coexistence of cancer. Even if extensive investigations are performed and a definitive diagnosis remains elusive, surgery must be considered.

## Introduction

Colorectal cancer (CRC) is sporadically encountered along with diverticulitis. In Western countries, there is an increased risk of CRC in patients with diverticulitis [1]. Additionally, an increased risk of CRC has been demonstrated during short-term follow-up after diverticulitis treatment [2]. Therefore, colonoscopy is recommended at 6-8 weeks after diverticulitis treatment [3,4].

However, the diagnosis of coexisting CRC at the onset of diverticulitis is challenging. Imaging studies often yield analogous findings for malignancy and diverticulitis, hindering their differentiation [5]. Thus, when diagnosing diverticulitis, there is a need to consider the potential coexistence of cancer and tailor treatment strategies accordingly.

We report the case of a patient in whom diverticulitis management was problematic and who had suspected concomitant CRC. The condition was completely resolved after prompt surgical intervention.

## Case presentation

**Figure 1:**
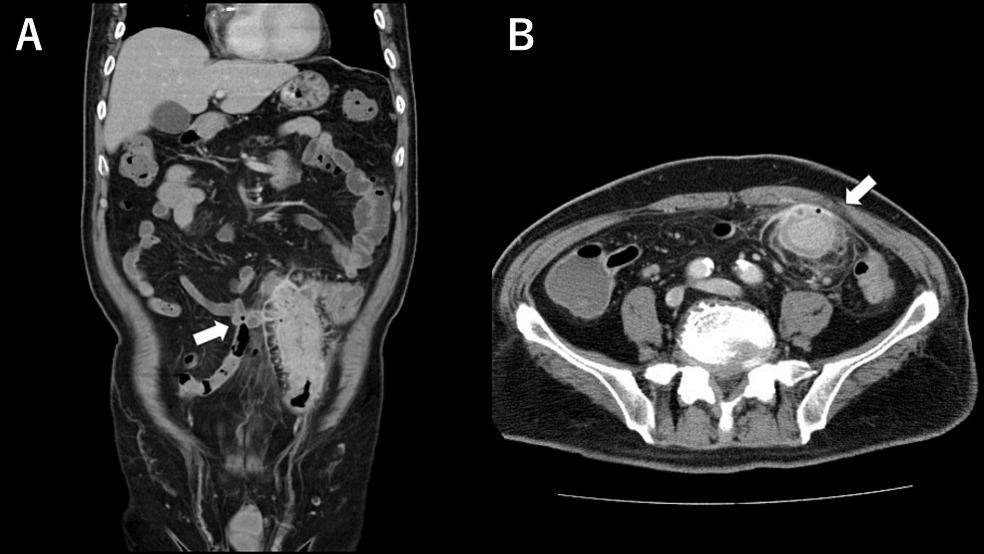
Coronal (A) and axial (B) sections of the abdomen and pelvis The sigmoid colon shows circumferential wall thickening and luminal stenosis with increased density in surrounding adipose tissue, along with an abscess. The sigmoid colon is in contact with the small intestine and abdominal wall (white arrows)

An 85-year-old male presented to a local clinic with lower left abdominal pain. Based on the suspicion of diverticulitis, oral antibiotic treatment was initiated. However, the patient’s symptoms did not improve, and he was admitted to our hospital for further evaluation and treatment. The patient had a history of diabetes mellitus. On admission, a physical examination revealed a body temperature of 38.3℃ and a palpable, firm mass in the lower left abdomen. Laboratory tests revealed an elevated white blood cell count (13,500/μL) and C-reactive protein level (10.4 mg/dL). Tumor marker levels were within normal limits. Abdominal computed tomography showed multiple diverticula in the sigmoid colon with circumferential wall thickening, as well as contrast enhancement and increased density in the surrounding adipose tissue. The wall structure was intact, but an abscess with air was present adjacent to the small bowel and abdominal wall (Figure 1A, 1B). A colonoscopy revealed a circumferential stricture in the sigmoid colon that could not be passed by the endoscope. No protruding lesion was observed on the mucosal surface. Biopsy results were not suggestive of malignancy. Barium enema examination revealed a 10 cm stricture without fistulae in the sigmoid colon.

Based on the above findings, the patient was diagnosed with sigmoid diverticulitis with abscess. Despite fasting and conservative treatment involving antibiotics, the inflammation recurred. Abdominal computed tomography showed no improvement in the diverticulitis, and conservative treatment was considered ineffective. A surgical approach was used, based on the possibility of CRC indicated by the palpable mass. During surgery, the sigmoid colon displayed adherence to a portion of the small bowel and abdominal wall. Sigmoid colectomy, partial small bowel resection, combined abdominal wall resection, and standard local lymph node dissection were performed in accordance with recommendations for the surgical treatment of CRC. The gross examination of the resected specimen revealed 10 cm circumferential wall thickening and sclerosis. The most constricted portion of the bowel exhibited significant bowing, which hindered the visualization of the mucosa, but no palpable tumor was apparent.

**Figure 2:**
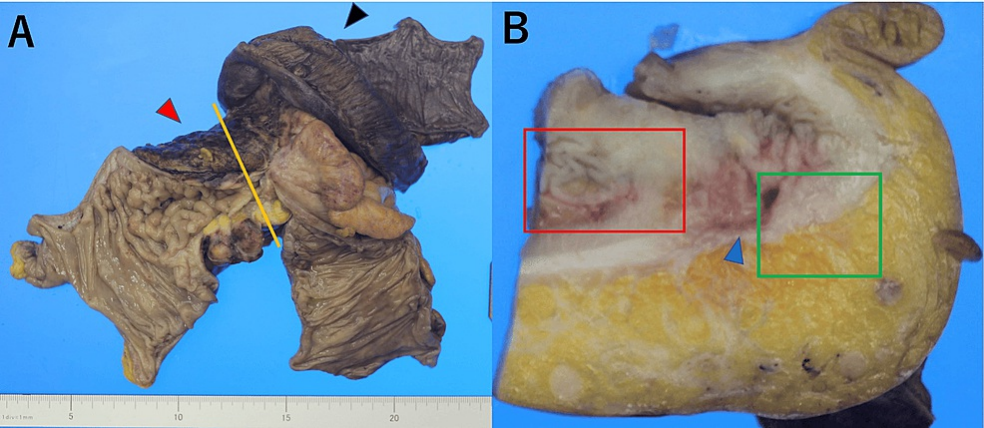
Macroscopical finding of resected specimen (A) and cross-section with yellow lines in A (B) The black arrowhead indicates a small bowel. The red arrowhead indicates the abdominal wall (A). The macroscopic image shows the fusion of intestinal loops because of inflammation, forming a single mass (B). A reddish lesion is present in the invaginated submucosa (blue arrowhead)

**Figure 3:**
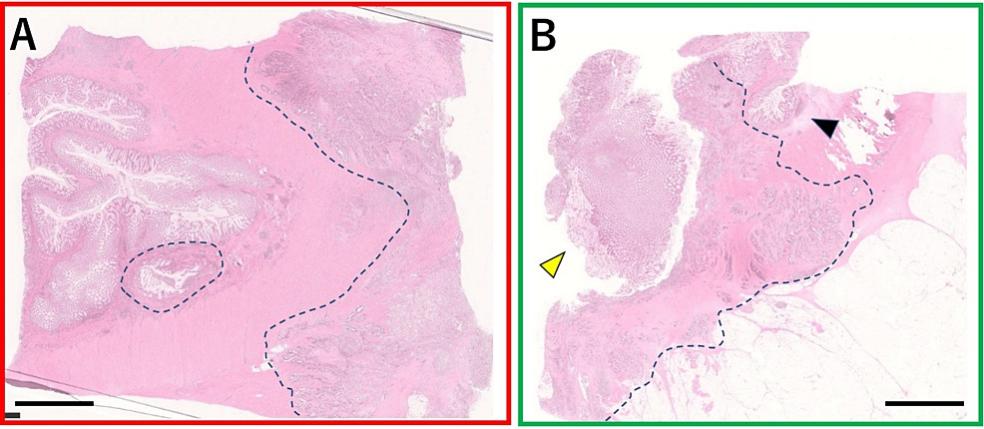
Pathologic images Blue dashed lines indicate the tumor area. The infiltration of the muscular layer and the presence of cancer cells extending to the submucosal layer are shown (A). Surrounding the cancer, diverticula (black arrowhead) and detached regenerative epithelium (yellow arrowhead) are evident (B). Scale bars: 5 mm

Histopathologic examination revealed that the most constricted portion of the sigmoid colon had overlapping and folded intestinal loops that had fused because of inflammation (Figure 2A, 2B). Within this area, a 33 mm adenocarcinoma was identified. The predominant histologic type was moderately differentiated tubular adenocarcinoma. Invasion reached the resected abdominal wall, but the resection margins were negative. Cancer cells exhibited slight exposure to the mucosa; tumor cells were observed in a portion of the invaginated intestine and from the muscular layer to the submucosa of the folded and overlapping separate intestinal loops (Figure 3A, 3B). Detached regenerated epithelium was present on the surface of the invaginated mucosa. Diverticula were observed in the vicinity of the tumor, suggesting that the invaginated mucosal structure had been formed by diverticulitis and the cancer had developed at this site. No lymph node metastasis or small bowel invasion was observed. The final diagnosis was sigmoid colon cancer with concomitant diverticulitis, stage IIC (T4bN0M0), according to the eighth Union for International Cancer Control (UICC) classification. The postoperative course was uneventful. Because of the patient’s advanced age, no adjuvant chemotherapy was administered after surgery. No recurrence was observed at one year postoperatively.

## Discussion

The one-year incidence of CRC in patients with diverticulitis is 1.9% [6], and diverticulitis is associated with an increased risk of CRC [1,7-9]. Diverticulitis and CRC occur predominantly in older adults; population aging is leading to an increasing frequency of cases involving both conditions [2,6,10]. The characteristics of CRC associated with diverticulitis include an increased rate of development in the sigmoid colon, diagnosis related to manifestations such as perforation or significant stricture, and a significant proportion of cases classified as T3 or higher according to the eighth UICC classification [11]. Therefore, it is important to consider the potential coexistence of CRC when diagnosing sigmoid diverticulitis in an older patient.

Reports of CRC arising from diverticular mucosa are scarce, presumably because of the difficulty in confirming colonic diverticular mucosa-related carcinogenesis based on pathologic findings. CRC arising within diverticula tends to be more advanced because the lack of a muscular layer facilitates serosal penetration [12]. Mechanistic analysis suggests that chronic inflammation from diverticulitis contributes to cancer development [12,13]. In the present case, multiple diverticula surrounded the malignant lesion, which formed at a site covered by a stricture and diverticulitis-related abscess. Diffuse infiltrative-type CRC was also present, which is atypical. Similar to inflammatory bowel disease-associated cancer, diverticulitis as a chronic inflammatory condition may have influenced cancer development and progression in the present case. The pathologic findings revealed cancer originating from the invaginated mucosa, with cancer cells present in the submucosa of a distinct bowel loop. Based on this evidence, we suspected that CRC had originated from the diverticular mucosa in this patient.

The preoperative diagnostic rate of CRC associated with diverticulitis is strikingly low, at 37% [11]. This low rate is because tumor cells predominantly infiltrate the submucosal layer, proliferate diffusely, and have limited exposure to the mucosal surface. Additionally, diverticulitis causes luminal narrowing related to medial muscular layer thickening, fibrosis, and mucosal overgrowth, hindering satisfactory assessment by endoscopy. On imaging, the intestinal wall structure appears to be preserved in diverticulitis, whereas it typically deteriorates in cancer [14,15]. However, in the present case, the presence of luminal stenosis, the absence of tumor cells on the mucosal surface (determined by pathologic examination), and the observation of circumferential wall thickening with preserved layer structure (determined by computed tomography) made preoperative diagnosis challenging, despite the suspicion of concurrent cancer.

In cases where diverticulitis does not improve with conservative treatment, concealed cancer is a possibility. Several predictive factors for diverticulitis have been reported [16]; however, the presence or absence of concomitant cancer is a critical consideration. When diverticulitis and CRC coexist, the resulting protracted diverticulitis may cause the cancer to be missed. This presumably occurs because inflammation persists in areas containing cancer cells, where mucosal regeneration is impeded. In the present case, the pathologic findings supported the hypothesis that inflammation was prolonged by the cancer-mediated inhibition of mucosal regeneration, as demonstrated by the presence of detached regenerative mucosa in the region containing cancer cells.

Interval surgery is considered beneficial in the management of diverticulitis [17]. However, in cases of refractory diverticulitis, it is important to consider the possibility of concurrent cancer. In the present case, curative resection was achieved in accordance with the recommendations for CRC surgery. If extensive investigations are performed but no definitive diagnosis is made, surgical intervention must be considered.

## Conclusions

The diagnosis and treatment of colorectal cancer associated with diverticulitis are complex and challenging. In refractory diverticulitis with abscess, it is important to consider the possibility of concealed malignancy. However, preoperative diagnosis is typically difficult; thus, it is important to suspect cancer in older patients with diverticulitis and select treatment strategies accordingly.
